# Correction: Site-specific DNA functionalization through the tetrazene-forming reaction in ionic liquids

**DOI:** 10.1039/d2sc90102a

**Published:** 2022-05-30

**Authors:** Seiya Ishizawa, Munkhtuya Tumurkhuu, Elizabeth J. Gross, Jun Ohata

**Affiliations:** Department of Chemistry, North Carolina State University Raleigh NC 27695 USA johata@ncsu.edu

## Abstract

Correction for ‘Site-specific DNA functionalization through the tetrazene-forming reaction in ionic liquids’ by Seiya Ishizawa *et al.*, *Chem. Sci.*, 2022, **13**, 1780–1788, https://doi.org/10.1039/d1sc05204g.

The authors regret that further investigations carried out following the publication of the original manuscript demonstrated that the phosphine-mediated bioconjugation reaction in ionic liquid produces a urea product instead of a tetrazene group. The authors have discovered that one of the key ^15^N NMR peaks at 300 ppm (mentioned in the Discussion: tetrazene formation by amine–azide coupling reaction section of the published manuscript) is an artifact from the intense solvent peak instead of the sample.^1^ The wrong characterization was also due to the mass similarity of the urea product and the tetrazene compound.

The authors have performed a number of experiments using NMR, X-ray crystallography, infrared spectroscopy, and mass spectrometry with different isotopically labelled reagents to confirm the urea structure. The details of the structure determination, as well as the reaction mechanism, are described in a manuscript on a preprint server.^2^ Corrected figures with the urea structure are shown below (Fig. 1 and 3). The ESI was modified accordingly and has been updated online (Fig. S13: the tetrazene structure was replaced with the urea structure. Fig. S15: an additional mechanism image of the urea formation has been included).

**Fig. 1:**
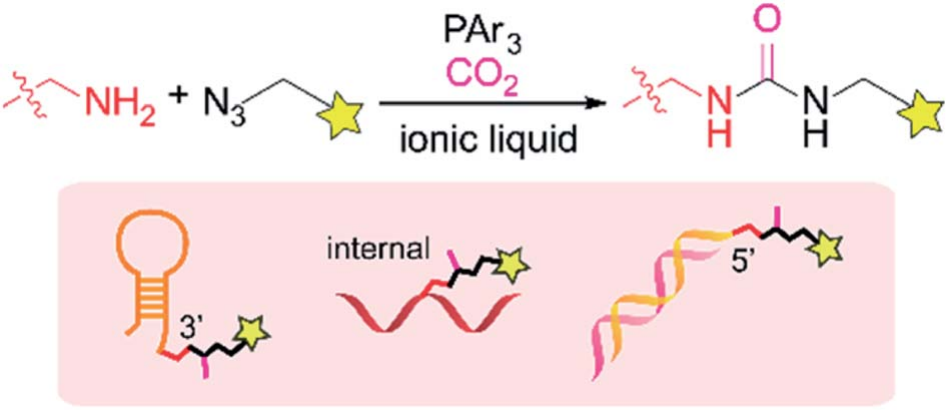
Site-specific urea-forming reaction on DNA substrates.

**Fig. 2:**
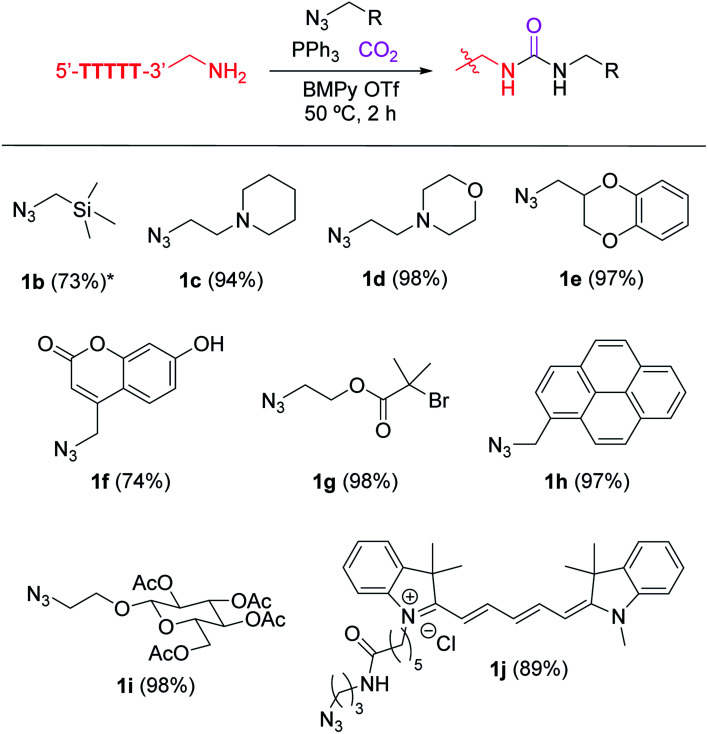
Urea-forming DNA bioconjugation with a variety of alkylazides. Modification reaction conditions: KHCO_3_ (20 mM), 5′-TTTTT-3′-alkyl-NH_2_ (0.2 mM), azide derivatives 1b–1j (7.5 mM), and PPh_3_ (20 mM) in BMPy OTf at 50 °C for 2 h. *Reaction was incubated overnight. Conversion in the parentheses were calculated based on matrix-assisted laser desorption/ionization (MALDI-MS) analysis. Conversion obtained by liquid chromatography is available in Fig. S2.

The authors believe that this correction does not change the overall conclusions of the published manuscript that are the ionic liquid-mediated site-specific bioconjugation of DNAs. The authors believe that the corrected manuscript remains of interest to the general chemistry readership of *Chemical Science* because the technology still offers the same capability for the site-specific labelling of DNAs with exquisite chemoselectivity and efficiency, enabled by a strategy to perform bioconjugation in nonaqueous media.

The authors have supplied a revised title, abstract and conclusion reflecting the aforementioned changes, which can be found below.

## Revised title

Site-specific DNA functionalization through the urea-forming reaction in ionic liquids.

## Revised abstract

Development of multiple chemical tools for deoxyribonucleic acid (DNA) labeling has facilitated wide use of their functionalized conjugates, but significant practical and methodological challenges remain to achievement of site-specific chemical modification of the biomacromolecule. As covalent labeling processes are more challenging in aqueous solution, use of nonaqueous, biomolecule-compatible solvents such as an ionic liquid consisting of a salt with organic molecule architecture, could be remarkably helpful in this connection. Site-specific chemical modification of unprotected DNAs through a urea-forming amine–azide coupling reaction using an ionic liquid was demonstrated. This ionic liquid-enhanced reaction process has good functional group tolerance and precise chemoselectivity, and enables incorporation of various useful functionalities such as biotin, cholesterol, and fluorophores. A site-specifically labeled oligonucleotide, or aptamer interacting with a growth factor receptor (Her2) was successfully used in the fluorescence imaging of breast cancer cell lines. The non-traditional medium-promoted labeling strategy provides an alternative design paradigm for future development of chemical tools for applications involving DNA functionalization.

## Revised conclusion

The ionic liquid-based urea-forming reaction has been successfully applied to the site-specific modification of unprotected DNA substrates. The high reaction efficiency at a desired location and high tolerance toward a variety of functional groups on azide and phosphine reagents could be of significant help in tailoring the technology to more specific applications. Thanks to the widespread use of azide–alkyne cycloaddition reactions in the chemistry and biology communities,^3^ there are numerous commercially available alkylazide reagents, and the current work can be readily adopted for diverse applications. The shelf-stable nature of the alkylazide and triarylphosphine reagents would also be practically helpful in this context. Persistent issues of common amine-targeting reagents originate from the reagent instability such as the hydrolytic decomposition of *N*-hydroxysuccinimide (NHS) ester reagents for the acylation reaction and the aerobic oxidation of aldehyde reagents used in the reductive alkylation reaction. Our initial success of ionic-liquid bioconjugation development for nucleotide substrates may serve to provide further access to untapped chemical labeling methodologies for preparation of nucleotide conjugates.

The Royal Society of Chemistry apologises for these errors and any consequent inconvenience to authors and readers.

## Supplementary Material
